# Methamphetamine signals transcription of IL1β and TNFα in a reactive oxygen species-dependent manner and interacts with HIV-1 Tat to decrease antioxidant defense mechanisms

**DOI:** 10.3389/fncel.2022.911060

**Published:** 2022-08-18

**Authors:** Liana V. Basova, Whitney Vien, Nikki Bortell, Julia A. Najera, Maria Cecilia Garibaldi Marcondes

**Affiliations:** ^1^San Diego Biomedical Research Institute, San Diego, CA, United States; ^2^The Scripps Research Institute, La Jolla, CA, United States; ^3^University of California San Diego, La Jolla, CA, United States

**Keywords:** Tat, methamphetamine, inflammation, reactive oxygen species, HIV, macrophage, NeuroHIV

## Abstract

Methamphetamine (Meth) abuse is a common HIV co-morbidity that is linked to aggravated Central Nervous System (CNS) inflammation, which accentuates HIV- associated neurological disorders, triggered both directly or indirectly by the drug. We used the well-established human innate immune macrophage cell line system (THP1) to demonstrate that Reactive Oxygen Species (ROS) immediately induced by Meth play a role in the increased transcription of inflammatory genes, in interaction with HIV-1 Tat peptide. Meth and Tat, alone and together, affect early events of transcriptional activity, as indicated by changes in RNA polymerase (RNAPol) recruitment patterns throughout the genome, via ROS-dependent and -independent mechanisms. IL1β (IL1β) and TNF α (TNFα), two genes with defining roles in the inflammatory response, were both activated in a ROS-dependent manner. We found that this effect occurred via the activation of the activator protein 1 (AP-1) comprising cFOS and cJUN transcription factors and regulated by the SRC kinase. HIV-1 Tat, which was also able to induce the production of ROS, did not further impact the effects of ROS in the context of Meth, but promoted gene activity independently from ROS, via additional transcription factors. For instance, HIV-1 Tat increased NFkB activation and activated gene clusters regulated by Tata box binding peptide, ING4 and IRF2. Importantly, HIV-1 Tat decreased the expression of anti-oxidant genes, where its suppression of the detoxifying machinery may contribute to the aggravation of oxidative stress induced by ROS in the context of Meth. Our results provide evidence of effects of Meth via ROS and interactions with HIV Tat that promote the transcription of inflammatory genes such as IL1β and TNFα.

## Introduction

Methamphetamine (Meth) is a highly addictive drug and a common comorbidity of Human Immunodeficiency Virus (HIV) infection. Meth aggravates neuroinflammation and cognitive disorders caused by HIV by acting on different cell types in the brain, and via a diversity of mechanisms, direct and indirect. These mechanisms include the action of the neurotransmitter dopamine (DA), which is highly increased by Meth in the brain and has effects on innate immune cells that are targets of HIV infection, affecting inflammatory markers and increasing viral spread (Basova et al., 2018). However, Meth acts on HIV targets in DA-independent ways, affecting particularly mitochondria and quickly resulting in high levels of Reactive Oxygen Species (ROS) (Yamamoto and Zhu, 1998; Brown and Yamamoto, 2003; Bortell et al., 2015). ROS affects gene transcription by modifying cysteine thiols that are critical for structure and function of molecules such as DNA, therefore, its effects, particularly on gene transcription, can be antagonized using scavengers such as N-acetyl cysteine (NAC) (Lee et al., 2007; Pedre et al., 2021). NAC has been shown by us to rescue and prevent important symptoms resulting from Meth exposure, such as hyperthermia (Sanchez-Alavez et al., 2013).

In physiological conditions, ROS balance is critical in signaling and metabolic pathways (Ray et al., 2012), and is maintained by anti-oxidant enzymes, such as superoxide dismutase (SOD), glutathione peroxidase (GP), glutathione (GSH) and catalase (CAT) (D’Autreaux and Toledano, 2007). On the other hand, ROS at higher levels may signal pathogenic patterns that include inflammatory cytokines, and other complications (Zuo et al., 2013; Cobb and Cole, 2015; Salisbury and Bronas, 2015; Raz et al., 2016). The influence of ROS on transcriptional activity caused by Meth in innate immune cells, and the interactions of these mechanisms with HIV, via its peptides, are not well understood.

In the CNS, Meth causes astrogliosis and microglial reactivity, with detectable alterations in important inflammatory cytokines, including tumor necrosis factor (TNF) (Gonçalves et al., 2010). Likewise, the Trans-activator of Transcription (Tat) is a HIV peptide with strong effects on gene transcription, largely due to its capacity to translocate to the host cell nucleus, acting epigenetically and as a transcription factor in host gene promoters (Veschambre et al., 1995; Kim and Panganiban, 1996; Rohr et al., 2003; Raha et al., 2005; Marban et al., 2011; Reeder et al., 2015; Tjitro et al., 2018; Basova et al., 2020). In addition, Tat also has the ability to increase ROS (Toborek et al., 2003; Capone et al., 2013). Understanding interactions between Meth- and Tat-induced mechanisms that are mediated by ROS or that bypass its contribution is critical to fully understand pathways increasing neuroinflammation in HIV + populations that use drugs.

Here, we have focused on the detection of effects of Meth on early gene activity that correlates positively with transcription in a ROS-dependent manner, and how HIV Tat interacts with ROS-dependent effects. We used a controlled *in vitro* macrophage cell line system, THP1 cells, to examine the impact of Meth on oxy-reduction and expression of anti-oxidant enzymes, as well as the effects of Meth-induced ROS on inflammatory gene activity, measured by recruitment pf RNA Polymerase (RNAPol) to promoters. We mimicked effects of HIV by using exposure to Tat, which is known to affect host gene transcription (Tjitro et al., 2018; Baek et al., 2020). We investigated the ability of Meth alone or combined with HIV Tat to lead to an unbalanced redox environment, where ROS upregulation signals the upregulation of sets of genes, including inflammatory markers. Meth and/or Tat were kept in cultures for short periods of time before analysis, in order to limit autocrine participation of released macrophage factors. We identified sets of ROS-responsive genes by comparing RNAPol recruitment in Meth- or Tat-treated cells, alone, combined, and in the presence of NAC. This approach allowed the identification of the earliest promoter activity resulting from ROS signaling, as well as the effects of Tat bypassing ROS regulation by promoting alternative transcriptional regulators. Genes that are relevant to HIV neuropathogenesis and found in drug-aggravated phenotypes, such as IL1β and TNFα, were found to be induced by Meth in a ROS-dependent manner and served as readouts to validate predictions and determine the associated transcriptional requirements in the context of Meth, with effects in the inflammatory transcriptional response.

## Materials and methods

### Cell cultures

Human THP1 cells were maintained using RPMI-1640 medium (Gibco, Thermofisher, Waltham, MA) supplemented with 10% fetal bovine serum (Hyclone, Cytiva, Marlborough, MA), 2mM Glutamine (Gibco), 100U/ml penicillin/streptomycin (Gibco), and 0.05mM beta-2-mercaptoethanol (Sigma-Aldrich, St. Louis, MO). Cultures were performed in 12 well plates (Corning, Corning, NY).

### Meth stimulation

Stimulation with (+)-methamphetamine hydrochloride (Meth, Sigma-Aldrich) was performed by adding 60uM of the drug in PBS to cultures containing 10^6^ cells/ml. The dose was selected based on previous optimization studies controlled for viability, and on concentrations that reach the brain in abuse, as previously described (Marcondes et al., 2010; Tjitro et al., 2018). The effects of the drug were examined on cells that were harvested at different time points following stimulation, for 15 minutes in ChIP-seq assays, and 2 hours for mRNA and protein measurements.

### Tat and NAC treatments

Recombinant HIV Tat (clade B, NIH AIDS Reagent Program, Bethesda, MD) was added to cells at 10ng/ml of PBS for the time points indicated. N-acetyl cysteine (NAC, Sigma-Aldrich) was used in cultures at 1uM, 30 minutes prior to or after the addition of Meth, as specified in the figure legends and results. The concentrations of Tat and NAC were previously optimized and described (Marcondes et al., 2010; Sanchez-Alavez et al., 2014, 2020; Najera et al., 2016; Tjitro et al., 2018).

### Other cell treatments

EGFR and Src inhibition was performed with 1 uM of Tyrphostin (Tyr, Sigma-Aldrich) and 1uM of PP2 (Sigma-Aldrich), respectively, added 30 min after Meth was used as stimulus.

### Hydrogen peroxide (H_2_O_2_) and superoxide (O_2_^–)^ measurements

Hydrogen peroxide production was measured in real time using Amplex Red Hydrogen Peroxide/Peroxidase Assay kit (Invitrogen, Waltham, MA), in an Infinite F500 luminometer (Tecan Systems, San Jose, CA). Superoxide was visualized on cell cultures using a treatment with 5 μM 2-hydroxyethidium (DHE, Sigma Aldrich, St. Louis, MO) freshly prepared prior to use from a 10 mM stock solution in DMSO. For that, 25 μl DHE stock solution was diluted in 50 ml Milli-Q pure H_2_O. After aspirating most of the media, and 130 ul of DHE staining solution was placed in each culture well and incubated for 20 min at room temperature and away from the light. The cells were washed twice in deionized water for just 1 min. Fluorescence imaging was performed immediately using an Axiovert 200 inverted microscope (Carl Zeiss) with Axio Vision software (version 4.8.1; Carl Zeiss). Fluorescence intensity was calculated in Fiji/ImageJ (National Institute of Health, United States) in tiff files were transformed into 8-bit, and manually thresholded to identify stained cells across all conditions. Measurement values were normalized to the area, in a minimum of six 60x magnification images for each condition in triplicate, and averaged.

### Protein extracts and western blots

Protein from cell cultures was extracted by lysis in radio-immunoprecipitation assay buffer (RIPA—Thermo Fisher Scientific, Waltham, MA) in the presence of Complete protease inhibitor cocktail tablets (Roche Molecular Biochemicals, Indianapolis, IN), following a wash with ice-cold PBS. The cells were scraped, transferred to a microfuge tube and spun at 10,000 rpm at 4°C for 10 min. The supernatant was transferred to a new tube and protein concentration was measured using a Pierce™ BCA Protein Assay Kit (Thermofisher). Protein was stored in −20°C until use. Ten micrograms of protein were loaded into lanes of SDS-PAGE electrophoresis (BioRad) in 4–20% gradient gels under reducing conditions. Transfer and immunodetection were performed as described (Bortell et al., 2017). Non-specific antibody binding was blocked using 5% non-fat dried milk for 1 h at room temperature. Immunoblotting was carried out with antibodies against cJun (clone 60A8), p-cJUN (cloneS63, Ser32), SRC (clone36D10), cFOS (clone 9F6), p-cFOS (cloneD82C12), NFkB p65 (clone D14E12), and IkB (cloneL35A5) (all Cell Signaling), p-SRC (clone 1246F, recognizes Y416), and pEGFR/ErB1 (clone Y1068) (both from R&D Systems, Minneapolis. MN), as well as anti-beta actin (clone 13E5, Cell Signaling, Danvers, MA), followed by secondary antibody HRP-conjugated anti-rabbit IgG (Novus, Littleton, CO), or anti-mouse (Cell Signaling). Blots were developed in film (Kodak, Rochester, NY) with 1:1 solution of Super Signal West Femto Chemiluminescent Substrate and Luminol/Enhancer (Thermo-Fisher Scientific, Rockford, IL). Bands were scanned, and band intensities were calculated in ImageJ (National Institute of Health, Bethesda, MD). Experimental bands were normalized to the intensity of beta-actin bands in each sample.

### Immunocytochemistry

The THP1 cells at the concentration of 10^6^/ml were stimulated with 50 nM of phorbol-12-myristate-13-acetate (PMA) for 24 h for macrophage differentiation prior to the stimulation with Meth, on poly-L-lisine (Sigma Aldrich)-treated 8-well glass chamber slides (Thermo Scientific), fixed with 4% paraformaldehyde for 20 min in the dark, and then washed with PBS. Wells were incubated with PBS containing 0.1% Triton X-100 for 15 min at room temperature, rinsed 3 times with PBS, and then blocked with 5 g/l Casein (Sigma Aldrich) in PBS, containing 0.5 g/l Thimerosal (Sigma Aldrich) for 1 h at room temperature. Primary antibodies against transcription factors were the same as in western blots, diluted in blocking solution and placed in wells for 2 h at room temperature. After rinsed 3 times with 1% blocking solution in PBS, secondary Alexa594-labeled donkey anti-rabbit or anti-mouse IgG (Thermo Fisher Scientific) were added for 2 h at room temperature, in the dark. After rinsing, 4′, 6-Diamidino-2-Phenylindole, Dihydrochloride (DAPI, Thermo-Fisher Scientific) was diluted to 300 ng/ml in 1% blocking solution for 10 minutes, in the dark. Cells were rinsed, maintained in PBS, and observed in a Nikon A1R laser-scanning confocal mounted onto a Nikon inverted Ti-E scope (Nikon, Melville, NY), and with a 20x PlanApo objective, 0.8NA (Nikon). Images were acquired using a NIS-Elements C software (Nikon). Fluorescence intensity was normalized against background (secondary antibody only). Image analysis was performed in Fiji/ImageJ (National Institute of Health, United States). For that, tiff files were opened and thresholded to identify stained cells from noise. For quantification, a binary mask was obtained from the DAPI image negative threshold and applied to the total transcription factor stained area. The translocation index was calculated as percentage of the total transcription factor stained measurement values within the nuclear area and derived from the difference between total and nuclear staining, as described (Tjitro et al., 2018).

### RNA polII chromatin immunoprecipitation

Early promoter activity with resulting changes in transcription rates was estimated as a function of RNA Polymerase II (RNAPol) occupancy by ChIP combined with Next-Generation sequencing (ChIP-Seq). For that, the cells were harvested, washed in ice cold PBS and the chromatin was crosslinked with 1% formaldehyde for 12 min, followed by glycine 0.125 M. The pellets were lysed with a lysis buffer containing 85 mM KCl, 0.5% NP40, 5 mM HEPES pH 8.0 and supplemented with cOmplete protease inhibitor cocktail (Sigma Aldrich), then incubated on ice for 15 min and centrifuged at 3500 g for 5 min. The pellet was resuspended in nuclear lysis buffer (10 mM EDTA, 1% SDS, 50 mM Tris–HCl, pH 8.1) at a ratio 1:1 (v/w), incubated on ice for 10 min, and stored at −80°C until use for RNAPol-ChIP. The chromatin extraction was performed using Chromatin IP DNA Purification kit (Active Motif, Carlsbad, CA). ChIP was performed using the ChIP-IT High Sensitivity Kit (Active Motif) with 30 μg of chromatin and 4 μg of antibody anti-RNA PolII Clone: 1F4B6 (Active Motif). ChIP Seq libraries were prepared and ChIP DNA was sequenced on the Illumina HiSeq using Active Motif Epigenetic Services. For analysis, data files were normalized to the same number of unique alignments without duplicate reads, which was 16 million sequence tags mapped to identify RNAPol binding sites. Intervals (peaks, “islands”) were determined using the SICER at a FDR1E-10 cutoff and a gap parameter of 600 bp to include promoters (which merges peaks located within 600 bp of each other into a single “island”). The number of intervals identified ranged from 9,775 in samples Meth + NAC to 14,124 in samples Meth + Tat. Data set was visualized in the UCSC Genome Browser.

### Systems biology and visualization tools

RNAPol peaks were averaged per group and transformed into fold change for visualization using local search features in GeneMANIA plugin (Mostafavi et al., 2008; Montojo et al., 2010; Warde-Farley et al., 2010) within Cytoscape v3.9.1 (Shannon et al., 2003) and reference Homo sapiens genome imported from BIOGRID_Organism (Stark et al., 2006; Oughtred et al., 2021). Transcription factor usage predictions were performed as previously described by us (Tjitro et al., 2018; Basova et al., 2021, 2022), using licensed TRANSFAC v2021.1 (Knuppel et al., 1994; Wingender et al., 1996) and iRegulon (Janky et al., 2014; Verfaillie et al., 2015) in Cytoscape v3.9.1.

### RNA extraction and qRT-PCR

Total RNA was isolated from the cells using Nucleospin RNA (Mackerey-Nagel, Germany), according to manufacturer’s instructions. Total RNA concentration was measured using the Nanodrop spectrophotometer and then used for reverse transcription using SuperScript III Reverse Transcriptase (Invitrogen, Waltham, MA). A screening of effects on oxidative stress pathway genes was performed using a Qiagen RT2 Profiler PCR array focused on Human Oxidative Stress (PAHS-065Y). Further validation was performed with primers purchased from Qiagen (Valencia, CA). PCRs were performed using RT2 SYBR Green ROX FAST Mastermix (Qiagen), in a 7900HT Fast Real-Time PCR System with Fast 96-Well Block Module (Applied Biosystems, Foster City, CA), with a SDS Plate utility v2.2 software (Applied Biosystems). The results were normalized to the average expression of GAPDH and 18S.

### TNFα and IL1β ELISA

Cell culture supernatants were collected at baseline, 15, 30, 60, 120, and 240 min following the stimulation with Meth, in the presence or absence of Tat and NAC, as well as Tyr and PP2. The cytokines were measured with TNF alpha Human ELISA kit (Invitrogen) and Human IL1 β/IL1 F2 Quantikine ELISA kit (R&D systems, Minneapolis, MN), following manufacturer’s protocols. Values were calculated based on standard curves.

### Statistical analysis

Group comparisons for individual genes across different conditions were performed using one-way or two-way ANOVA, followed by Bonferroni’s *post hoc* tests. The difference between the means was considered significant at *p* < 0.05. Tests were performed using Prism software (GraphPad Software, San Diego, CA, United States) for Macintosh.

## Results

### Both meth and Tat increase ROS production

**FIGURE 1:**
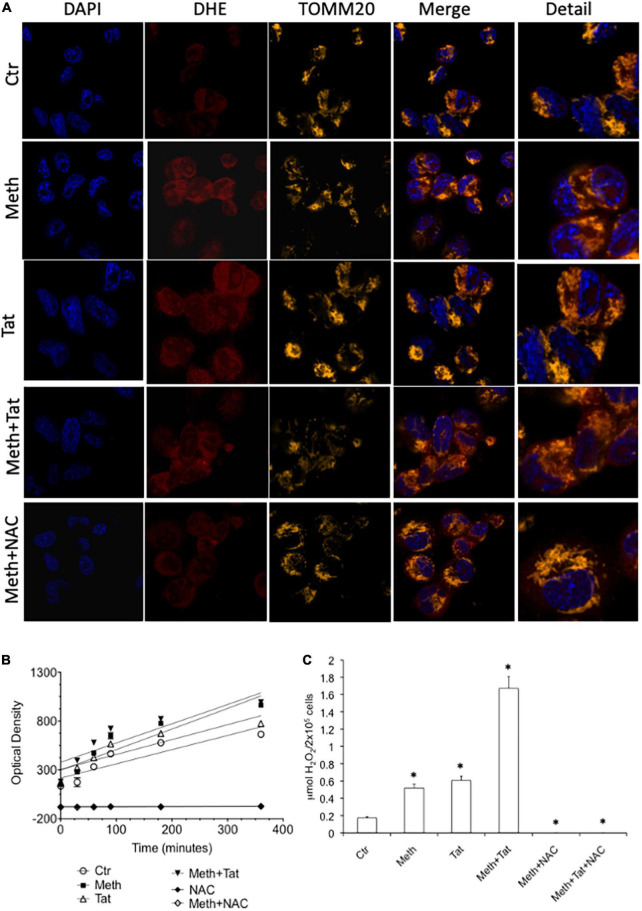
Meth induces ROS acutely in innate immune cells. The increase of ROS was examined in real time THP1 macrophages following the treatment with 60uM Meth, 10 ng/ml HIV Tat, or both together. The ROS scavenger NAC (1 uM) was added to cultures at time of stimulation. **(A)** Superoxide detection by DHE staining. DAPI was used for identifying the cell nucleus, and TOMM20 was used to identify mitochondria. Merging demonstrates that DHE is not solely associated to TOMM20 + mitochondria in macrophages, but it is also in the cell cytoplasm. **(B)** Amplex Red luminescence was used to detect H_2_O_2_ in real time for 360 minutes following stimulation. The graph shows the average of a triplicate, in one representative experiment, out of 5 performed. **(C)** The molarity of released H_2_O_2_ was calculated at 60 min, for all the conditions analyzed, *n* = 5 triplicates. *p < 0.05 compared to vehicle treated controls (Ctr).

We measured the ability of Meth and Tat to promote an increase of ROS Superoxide (O_2_^–^) and Hydrogen Peroxide (H_2_O_2_) and alter the expression of anti-oxidant and oxidative stress responsive genes in macrophages. Using the O_2_^–^ -sensitive red dye Dihydroethidium (DHE), we found that both Meth alone and with Tat increased the availability of this species detectable by confocal microscopy 15 min after exposure, and that DHE was largely co-localized with TOMM20 + mitochondria (Figure 1A). HIV Tat peptide alone caused increase of cytoplasmic superoxide, but when combined with Meth, it did not significantly change DHE labeling compared to Meth alone. The treatment with the anti-oxidant N-acetyl cysteine (NAC) decreased the DHE staining back to control levels in all conditions. The increase in superoxide detectable by DHE was correlated with higher levels of secreted H_2_O_2_ overtime in Meth-treated cells with or without Tat (Figure 1B). In addition, H_2_O_2_ production was completely abrogated by NAC (Figure 1B). Interestingly, the production of ROS following stimulation reached a plateau at 2 h, which persisted for several hours. At 60 min after stimulation, the absolute levels of H_2_O_2_ were calculated in the cultures to further estimate differences between Meth and Tat, alone and together, in their ability to modify the redox state of macrophages. This indicated that although superoxide was significantly increased with HIV Tat (Figure 1A), H_2_O_2_ levels were not different between Meth and Tat, but were significantly higher when Tat and Meth were together, compared to Meth or Tat alone (Figure 1C).

### Meth and Tat interactions cause a decrease in the transcription of antioxidant genes

**FIGURE 2:**
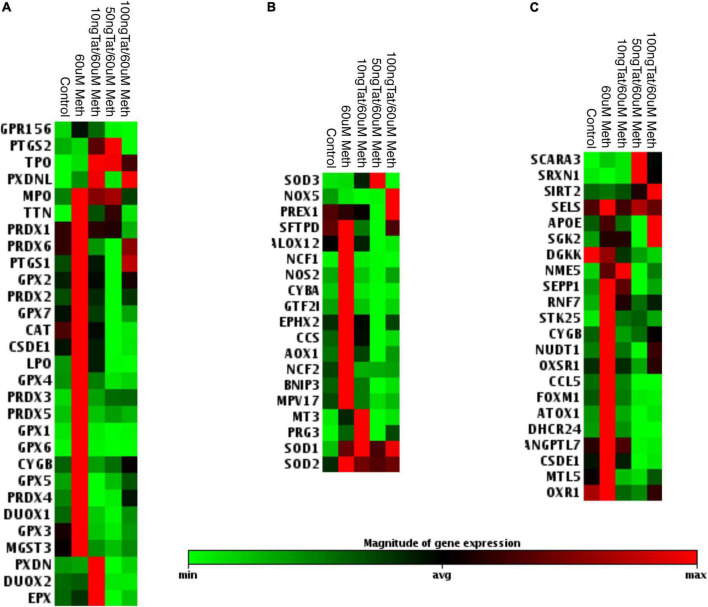
Effects of Tat on Meth-induced changes in the oxidative stress response in THP1 macrophages. The heat map shows the average transcription of oxidative stress genes 2 h after the stimulation with Meth alone and with Meth + HIV Tat peptide at the doses of 10, 50, and 100 ng/ml. Map shows significantly affected genes as per ANOVA *p* < 0.05. The genes were normalized by average of 3 independent experiments in duplicate and were organized by function categories and expression clustering. **(A)** Antioxidant pathways; **(B)** Reactive Oxygen Species Metabolism pathway genes; **(C)** Oxidative Stress Response Genes. Gene expression was measured using RT2 Profiler PCR array focused on Human Oxidative Stress.

**FIGURE 3:**
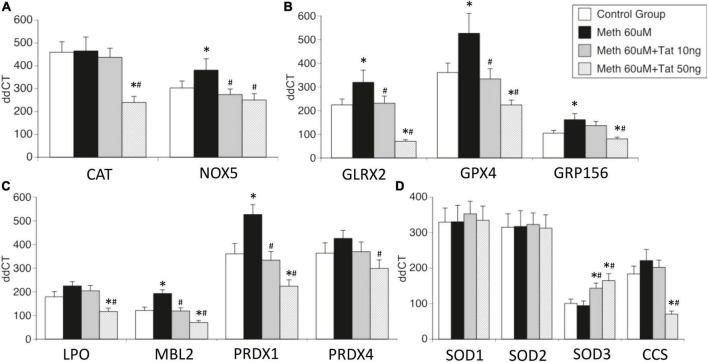
Effects of HIV Tat on Meth-induced transcriptional changes in oxidative stress-relevant genes. Validation was performed in selected genes most relevant in innate immune oxidative metabolism and detoxification in controls and in THP1 macrophages stimulated with Meth (60uM), in the presence and absence of HIV Tat at 10 and 50ng/ml. **(A)** Catalase CAT and NADPH oxidase 5 (NOX5); **(B)** Glutaredoxin 2 (GLRX2), Glutathione peroxidase 4 (GPX4, membrane associated), GABA b receptor (GRP156); **(C)** Lactoperoxidase (LPO), Mannose-binding lectin 2 (MBL2), Peroxiredoxins 1 and 4 (PRDX1 and PRDX4); **(D)** Superoxide dismutases 1, 2, 3 (SOD1, SOD2, SOD3), and Cu-chaperone for SODs (CCS). Values correspond to the mean ± SD of 3 independent experiments performed in duplicate. *p < 0.05 compared to vehicle stimulated controls, ^#^*p* < 0.05 compared to Meth only in Bonferroni’s *post hoc* test.

Due to the toxicity of ROS, the increase in antioxidant mechanisms is critical in conditions of oxidative stress to maintain a homeostatic balance both intracellularly, and in the environment (Halliwell, 1999; Virag et al., 2019). These mechanisms depend on different types of peroxidases, including catalase (CAT), superoxide dismutases (SOD), and other enzymes involved in the recycling of oxidized components, such as glutathione. We performed transcriptional analysis during treatment with Meth alone and during the addition of the HIV Tat peptide to identify changes in oxidative stress responsive genes. A panel of antioxidant and ROS metabolism pathway transcripts expressed by macrophages was measured 2 h after stimulation with Meth, in the presence or absence of HIV Tat (Figures 2, 3). We found that, for the most part, Meth stimulation was able to increase antioxidant and ROS metabolism gene transcription, but this increase was blocked by the addition of HIV Tat (Figure 2). This was observed in antioxidant genes (Figure 2A), in ROS metabolism genes (Figure 2B), as well as in genes that are responsive to oxidative stress (Figure 2C). In detail, only 4 antioxidant genes were increased by Meth + Tat but not by Meth alone, GPR156, PTGS2, TPO and PXDNL (Figure 2A), and MPO was increased by both Meth alone and together with Tat. All other antioxidant genes were increased by Meth but suppressed by the combination of Meth + Tat at all doses. These included TTN, Peroxiredoxins (PRDX) PRDX1, PRDX2, PRDX3, PRDX4, PRDX5, PRDX6, PTGS1 (COX1), Glutathione Peroxidases (GPX) GPX1, GPX2, GPX3, GPX4, GPX5, GPX6, GPX7, Catalase (CAT), CSDE1, LPO, CYGB, DUOX1, and MGST3 (Figure 2A). PXDN, DUOX2 and EPX were increased only by the combination of Meth and the lowest concentration of Tat (10ng/ml), but not by the other conditions (Figure 2A). In the reactive oxygen metabolism pathway (Figure 2B), we observed an increase in the superoxide dismutases SOD1 and SOD2 by Meth alone as well as by the combination of Meth + Tat at any dose, and an increase in SOD3, NADPH oxidase (NOX5) and PREX1 only by Meth + Tat at higher doses (Figure 2B). On the other hand, and similar to antioxidant genes, Meth alone increased a number of genes in this pathway, while Tat repressed the effect of Meth. These genes were STFPD, ALOX12, NCF1, NOS2, CYBA, GTF2I, EPHX2, CCS, AOX1, NCF2, BNIP3, and MPV17. MT3 and PRG3 were increased only by Meth + Tat at the lowest dose (Figure 2B). We also examined genes involved in the oxidative stress response, including oxygen transporters and pathway signatures (Figure 2C). A similar trend was observed, with genes such as SCARA3, SRXN1, and SIRT2 being increased by the combination of Meth and Tat at high doses, but not by other conditions, and all other genes being increased or maintained at high levels by Meth but suppressed by the combination of Meth and Tat. These genes included SELS2, APOE, SGK2, DGKK, NME5, SEPP1, RNF7, STK25, CYGB, NUDT1, OXSR1, CCL5, FOXM1, ATOX1, DHCR24, ANGPTL7, CSDE1, MTL5, and OXR1 (Figure 2C). The blockage of the majority of Meth effects by Tat was stronger at 50 ng/ml compared to 10 ng/ml and to 100 ng/ml indicating an optimal dose effect. In subsequent assays, we focused on lower doses of Tat that are more physiologically relevant.

The effects of Tat on changes triggered by Meth were further validated in isolated genes such as CAT and NOX5 (Figure 3A), Glutaredoxin, Glutathione peroxidase and GABA b receptors (Figure 3B), Lactoperoxidase, Mannose-binding protein and Peroxiredoxins 1 and 2 (Figure 3C), as well as in Cu chaperone (Figure 3D). Interestingly, in validation experiments SOD1 and SOD2 were not affected by Meth in the presence or absence of Tat, and SOD3 was enhanced by Meth and Tat in a dose-dependent manner (Figure 3D). Overall, the data indicates that genes involved in mechanisms that facilitate H2O2 production and enhance oxidative stress may remain unchanged and active, but genes involved in detoxification of ROS are transcriptionally decreased by the interaction between Meth and soluble HIV peptides, suggesting that in the context of Meth and HIV interactions, damage by oxidative stress may be a factor leading to disorders.

### Meth- induced inflammatory gene transcription occurs *via* ROS signaling and is enhanced by HIV Tat

**FIGURE 4:**
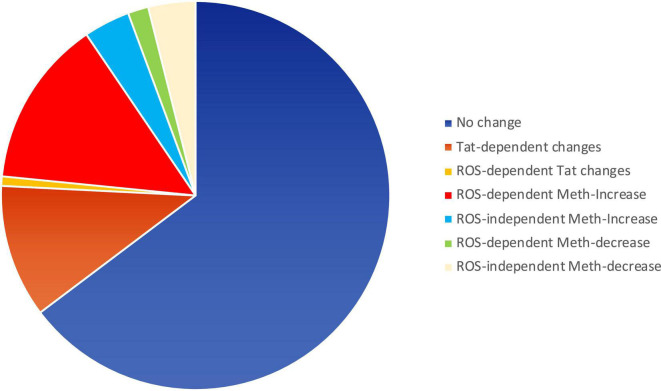
Pie chart showing the relative impact of Meth and Tat on RNAPol recruitment to gene promoters in macrophages and the effects of ROS. RNAPol peak values from ChIP were obtained in THP1 cells treated with Meth (60 uM) or Tat (10 ng/ml) for 15 min, in the presence or absence of NAC (1 uM).

ROS may serve as secondary messengers in signaling intracellular regulation of gene transcription (Turpaev, 2002; Virag et al., 2019), by activating protein kinases, phosphatases, and transcription factors (Turpaev, 2002). We tested whether ROS induced in the context of Meth, with redox consequences that are enhanced by Tat, can be responsible for increasing the transcription of inflammatory genes. For that, we examined changes in early events associated with active transcription, indicated by the changes in RNA Polymerase II (RNAPol) recruitment to promoter transcription starting sites (TSS) throughout the genome. Chromatin immunoprecipitation (ChIP) to identify the frequency of RNAPol binding was performed as early as 15 min following macrophage exposure to Meth, with or without Tat. The time point of 15 min was chosen to reflect changes that were immediately due to stimuli prior to the upregulation of secondary factors such as cytokines. In this system, we addressed the contribution of ROS by comparing cells treated with Meth and/or Tat with the effects of a ROS scavenger NAC, added to cultures 30 min prior to Meth, for abrogating H_2_O_2_ production, as seen in Figure 1. In the analysis, we focused on gene promoter intervals that showed increased RNAPol recruitment by Meth, but that was prevented by the addition of NAC treatment, to distinguish pro-transcriptional promoter activity that was associated with ROS. The majority of the detectable RNAPol on promoters was not affected by these stimuli, which corresponded to 8,292 genes that experienced no change. Tat modified RNAPol recruitment in 1,418 genes. Of the 2,279 genes that increased RNAPol recruitment due to Meth, 1,786 did it so in a ROS-dependent manner (prevented by NAC). Of the 722 genes that decreased RNAPol recruitment as a result of Meth, 503 were dependent of ROS. Figure 4 schematically indicates the large contribution of ROS to changes in RNAPol recruitment that are triggered by Meth in innate immune macrophages.

With Meth as a stimulus, we found 2146 genes with detectable increase in RNAPol recruitment to promoter regions (> 1.5-fold, *p* < 0.05). Of these, 1,729 genes had RNAPol promoter recruitment prevented by NAC, indicative of critical ROS signaling contributions. In addition, 225 genes had a decrease in RNAPol accumulation caused by Meth, of which 219 had their downregulation prevented or recovered by NAC. In order to identify ROS-dependent patterns that are dependent on Meth and enhanced by HIV, we used systems biology to examine RNAPol recruitment changes that were prevented by NAC. These genes were functionally annotated (Kyoto Encyclopedia of Genes and Genomes, KEGG analysis resource), to metabolic disease (*p* = 0.0004), chemical dependence (*p* = 0.0005), hematological (*p* = 0.001) and to aging processes (*p* = 0.08), all relevant in the context of HIV and substance use.

**FIGURE 5:**
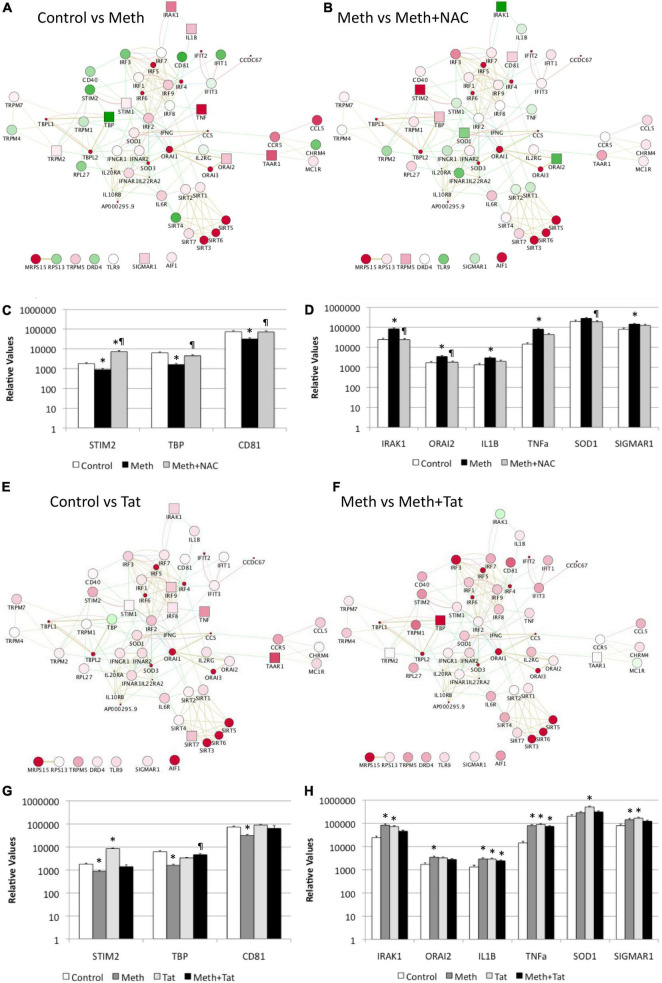
Effect of Meth-induced ROS and HIV Tat on RNAPol recruitment to promoter intervals in macrophages, and on transcription of selected genes. RNAPol peak values from ChIP, in THP1 cells treated with Meth (60 uM) for 15 min. Patterns were identified in Cytoscape, *via* GeneMania and JActiveModules. The highest score node was selected for further analysis. **(A)** The behavior of the highest score gene cluster was compared between Meth and control, vehicle-treated cells. **(B)** The behavior of the highest score gene cluster was compared between Meth + NAC and Meth alone-treated cells. Shades of red indicate levels of upregulation, and shades of green show levels of downregulation of RNAPol accumulation/promoter activity. Squares correspond to changes that were statistically significant. Validation was performed by measuring mRNA transcription with qRT-PCR, 2 h after treatments. **(C)** Transcription of genes with RNAPol activity decreased by Meth and reverted by NAC. **(D)** Transcription of genes with RNAPol activity increased by Meth and reverted by NAC. The impact of Tat (10ng/ml) was evaluated in the genes in the validated gene cluster. *p < 0.05 compared to control. ℜ*p* < 0.05 compared to Meth. **(E)** The behavior of the highest score gene cluster was compared between Tat and control, vehicle-treated cells. **(F)** The behavior of the highest score gene cluster was compared between Meth and Meth + Tat-treated cells. Shades of red indicate levels of upregulation, and shades of green show levels of downregulation of RNAPol accumulation/promoter activity. Squares correspond to changes that were statistically significant. Validation was performed by measuring mRNA transcription with qRT-PCR, 2 h after treatments. **(G)** Transcription of genes with RNAPol activity decreased by Meth and reverted by Tat. **(H)** Transcription of genes with RNAPol activity increased by Meth and by Tat. The impact of Tat (10ng/ml) was evaluated in the genes in the validated gene cluster. *p < 0.05 compared to control. ℜ*p* < 0.05 compared to Meth.

In Cytoscape, we applied JActiveModules clustering strategies to reveal orchestrated behaviors in gene networks indicative of biological processes that are affected by ROS in the context of Meth, and aggravated by HIV Tat (Figures 5A,B,E,F). The highest score gene network, connected via protein-protein interactions (pink connectors), shared protein domains (yellow connectors), co-localization (blue connectors), and genetic interactions (green connectors) was functionally annotated (KEGG) to the Toll-like receptor pathway (*p* = 3.4E-10), Cytokine-cytokine receptor interaction (*p* = 1.5E-6), Jak-Stat signaling pathway (*p* = 6.6E-4), Cytosolic DNA-sensing pathway (*p* = 2.1E-3), and Natural killer mediated cytotoxicity (*p* = 2.4E-2), and transmembrane and membrane-associated molecules (*p* = 5.7E-6). Interestingly, Meth significantly downregulated RNAPol recruitment to the TATA box binding peptide (TBP) promoter, shown as a green square. On the other hand, Meth significantly increased activity at inflammatory genes such as IRAK1, IL1β, TNFα, and ORAI1, as well as TAAR1 and Sigma1R (Figure 5A). NAC significantly reverted the effects of Meth on TNFα, ORAI1, IRAK1 and IL1β gene activity, and also decreased RNAPol in the SOD1 promoter. NAC did not prevent the Meth-induced increase in TBP and TAAR1, when compared to Meth alone (Figure 5B). HIV Tat upregulated several genes but had no effect on the RNAPol recruitment to the TBP promoter (Figure 5E). However, although the effects of Meth and Tat interactions were not additive of synergistic, Tat in the context of Meth highly upregulated TBP gene activity (Figure 5F), suggesting the role of this transcription factor in aggravated pathogenesis, and as described by us (Tjitro et al., 2018).

**FIGURE 6:**
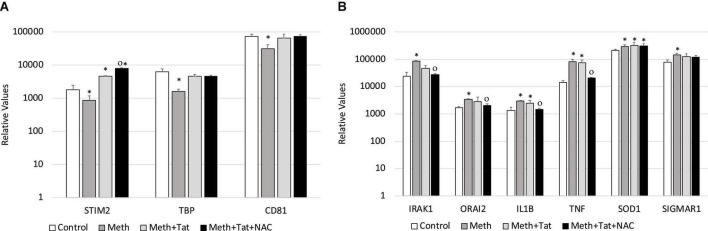
Transcriptional changes that are induced by Tat in the context of Meth are both ROS-dependent and ROS-independent. The ROS-dependent effects of Tat on gene transcription, in the context of Meth, were tested by qRT-PCR in cultures that were treated with Meth, Tat and with NAC, 2 h after treatments. **(A)** Transcription of genes with RNAPol activity decreased by Meth and reverted by Tat, with no effect of NAC in the context of Tat. **(B)** Transcription of genes with RNAPol activity increased by Meth with partial or no effects of Tat, but reverted by NAC in the context of Tat. Tat (10 ng/ml) and NAC (1 uM) were evaluated in the same genes. *p < 0.05 compared to control. ^O^*p* < 0.05 compared to Meth + Tat.

We validated our RNAPol ChIP findings by checking mRNA levels of specific genes associated with inflammation 2 h after Meth, in the presence and absence of NAC (Figures 5C,D) and in the presence and absence of HIV Tat (Figures 5G,H). Figure 4C illustrates genes with promoter activity decreased by Meth, but recovered by NAC, while Figure 3D shows genes with promoter activity increased by Meth and prevented by NAC in Meth-stimulated cultures. The effect of Meth-induced ROS on the downregulation of Tata-box binding protein (TBP), of the Ca + 2 sensor Stromal Interaction molecule 2 (STIM2) and of the signal transduction mediator CD81 was also noted at the transcriptional level (Figure 5C). Similarly, Meth significantly upregulated transcription of genes such as the Interleukin-1 Receptor-Associated Kinase 1 (IRAK1), the Calcium Release-Activated Calcium Modulator 2 (ORAI2), Interleukin 1 beta (IL1B), Tumor Necrosis Factor alpha (TNFα), in a ROS-dependent fashion, and the Meth-sensitive molecule Sigma Receptor 1 (SIGMAR1), and Superoxide Dismutase 1 (SOD1), as suggested by the ability of NAC to prevent the increase in transcription (Figure 5D). These data suggest that Meth affects the transcription of a number of pro-inflammatory genes in innate immune cells acutely, in a ROS-dependent manner. In spite of the effects of Meth and Tat on redox genes, we did not find a strong impact of interactions between Meth and Tat to alter the transcription of inflammatory genes using RT-PCR (Figures 5G,H). Tat alone increased the transcription of STIM2 (Figure 5G), IRAK1, IL1β, TNFα, and SIGMAR1 (Figure 5H), and Meth and Tat further increased the expression of TBP (Figure 5G). We tested whether there was redundancy in the ability of ROS to increase the transcription of these genes in the context of Meth and Tat interactions. For that, we examined whether the changes caused by the addition of Tat remained when cells were co-treated with NAC. Figure 6 shows that the effects of Tat alone and in the context of Meth on the transcription of IL1β and TNFα were prevented by NAC. This may suggest that the effects of Tat do not modify ROS-mediated signaling threshold but adds ROS-independent signaling to a number of genes, particularly genes suppressed by Meth in a ROS-dependent fashion. Tat also increases oxidative damage, introducing additional factors in pathogenesis, and a potential contribution of TBP.

### Changes in transcriptional regulation caused by meth- induced ROS and implications to inflammatory gene transcription

**FIGURE 7:**
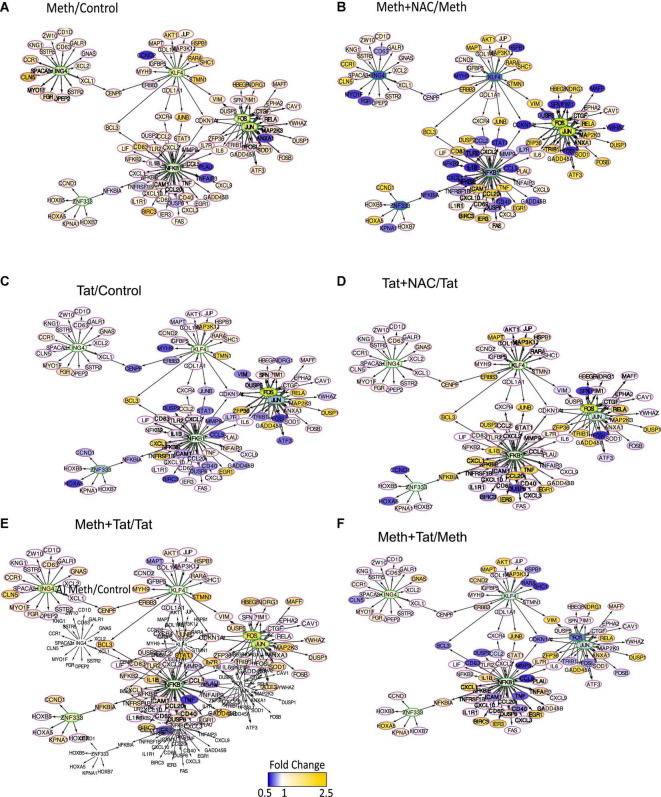
Transcription factor usage prediction in association with effects of ROS in macrophages stimulated with Meth. Visualization of the RNAPol promoter recruitment changes in THP1 cells 15 min after stimulation with **(A)** Meth (60 uM) in relation to vehicle control, or **(B)** Meth + NAC in relation to Meth alone, **(C)** HIV Tat (10ng/ml) in relation to Control, **(D)** Tat + NAC in relation to Tat, **(E)** Meth + Tat in relation to Tat alone and **(F)** Meth + Tat in relation to Meth alone. TRANSFAC prediction was confirmed using merged iRegulon-predicted transcription factor megatargetomes, for visualization of significant (*p* < 0.05) relative fold changes and cluster regulation. Predicted transcription factors, cFOS and cJUN (AP1), NFkB, ZNF333, KLF4, and ING4 positioned in the center of regulated gene clusters, and RNAPol recruitment to gene promoters is indicated by node colors, decreased in tones of blue and increased recruitment in tones of yellow.

We used iRegulon and TRANSFAC to predict changes in transcription factor usage associated with ROS in the context of Meth, by examining the frequency of transcription factor binding motifs in genes upregulated by Meth and inhibited by the co-treatment with NAC (Figure 7). NFkB, JUN and FOS (AP1) binding domains were most frequently identified, with respectively 1070 and 788 identified binding motifs. These were followed by 673 binding motifs for KLF4, 597 for ING4 and 576 for ZNF333. Together, these transcription factors were predicted to regulate overlapping gene networks that had enhanced RNAPol recruitment activity in the context of Meth (Figure 7A), but drastically decreased by the co-exposure of cells to the ROS scavenger NAC (Figure 7B). The majority of the inflammatory genes that are relevant in pathogenesis appeared to be regulated by FOS/JUN, which form the AP1 complex, and/or by NFkB (Figure 7). Tat alone was less effective at activating genes regulated by AP1 and NFkB, with many genes showing decreased RNAPol recruitment, in tones of blue (Figure 7C). Interestingly, Tat combined with NAC increased RNAPol activity in a number fo the genes with activity repressed by Tat, suggesting a contribution of ROS to the effects of Tat, although to the opposite direction of Meth alone (Figure 7D). When Meth and Tat were together in the cultures, gene activity particularly in genes regulated by AP1 was higher than in Tat alone (Figure 7E), confirming that Meth activates these predicted transcription factors. On the other hand, the effect of Tat and Meth compared to Meth alone indicated lower activity in AP1-regulated genes (Figure 7F). This result indicated that Tat activates other transcription factors.

**FIGURE 8:**
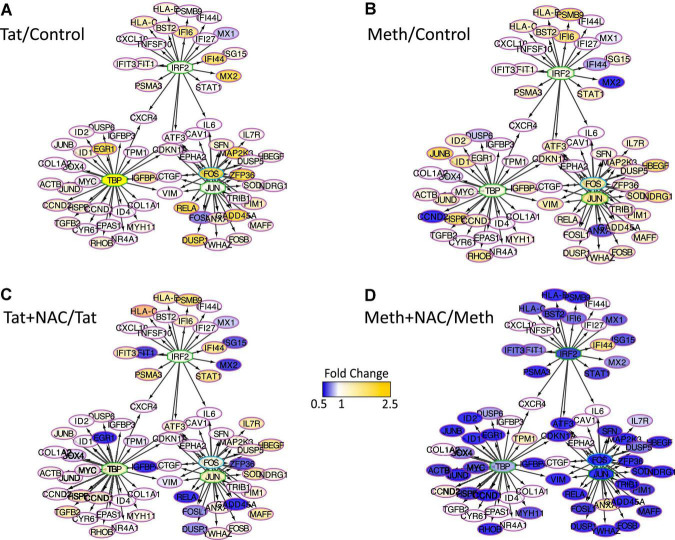
Transcription factor predictions derived from changes in RNAPol caused by Tat, overlapping and interacting with the effects of ROS. Visualization of the RNAPol promoter recruitment changes in THP1 cells 15 min after stimulation with **(A)** HIV Tat (10 ng/ml) in relation to control, **(B)** Meth (60 uM) in relation to vehicle control, **(C)** Tat + NAC in relation to Tat, and **(D)** Meth + NAC in relation to Meth alone. TRANSFAC prediction was confirmed using merged iRegulon-predicted transcription factor megatargetomes, for visualization of significant (*p* < 0.05) relative fold changes and cluster regulation. IRF2, TBP and cJUN/cFOS (AP1) positioned in the center of regulated gene clusters, and RNAPol recruitment to gene promoters is indicated by node colors, decreased in tones of blue and increased recruitment in tones of yellow.

The changes in RNAPol significantly induced by Tat were used to predict transcription factor usage, as previously described (Tjitro et al., 2018), overlapping and interacting with the effects of ROS (Figure 8). Using iRegulon, we have confirmed that TBP, IRF2, cJUN, and cFOS were predominantly used by Tat, which increased activity in genes regulated by these factors (Figure 8A). Meth also increased gene activity regulated by IRF2 and TBP, in addition to AP1 (Figure 8B). However, while NAC drastically decreased the changes caused by Meth in this network (Figure 8D), it did not suppress the effect of Tat, particularly under control of TBP and IRF2 (Figure 8C), suggesting that the actions of Tat on transcriptional regulation are mostly ROS-independent, or co-regulated by transcription factors or binding sites that are not affected by the redox environment.

### ROS-induced changes in transcription factor dynamics regulate transcription of key inflammatory genes upregulated by meth: Effects on IL1β and TNFα

**FIGURE 9:**
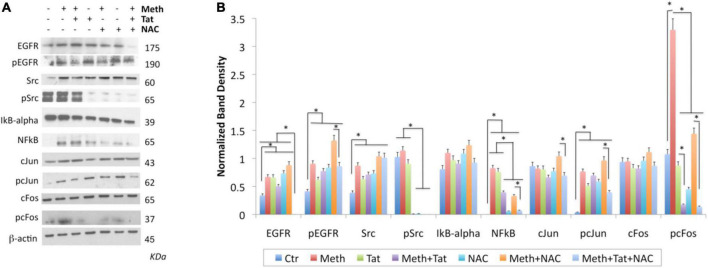
Changes in transcription factors activated by Meth. Western blot was used on protein extracts of THP1 cells stimulated for 2 h with Meth (60 uM), Tat (10 ng/ml) and/or NAC (1 uM) to examine changes in the levels of EGFR, pEGFR, SRC, pSRC, IkB-alpha, NFkB, cJUN, pcJUN, cFOS, and pcFOS, using specific antibodies. **(A)** Representative western blots showing all the analyzed transcription factors and the examined conditions. Molecular weight of each band is shown on the right side of the panel. **(B)** B-actin- normalized band density calculated in Fiji Image J, from 3-4 independent experiments. *p < 0.01 in indicated comparisons. ANOVA followed by Bonferroni’s test.

**FIGURE 10:**
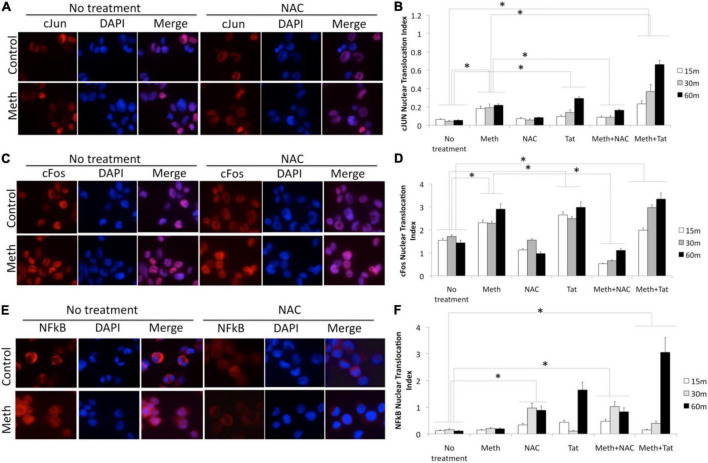
Translocation of cJUN, cFOS and NFkB by Meth, effects of ROS and of Tat. Translocation was measured in THP1 cells stimulated with Meth (60 uM), Tat (10ng/ml) and/or NAC (1 uM), by immunocytochemistry using specific antibodies to pan-cJUN **(A,B)**, pan-cFOS **(C,D)** and NFkB p65 **(E,F)**, and a secondary AlexaFluor 594-conjugated antibody. Secondary antibody controls were negative (not shown). Representative immuno-cytochemistry in vehicle or Meth and/or NAC treatment, showing cJUN **(A)** cFOS **(C)**, and NFkB **(E)**. The nucleus was identified with DAPI staining. Translocation index was calculated for cells treated with Meth, and/or Tat, and/or NAC, 15, 30, and 60 min after stimulation, for detection of differences in the translocation of cJun **(B)**, cFos **(D)** and NFkB **(F)**. The index was obtained using measurement values obtained in Fiji ImageJ, in 5 images taken from each well, with all conditions performed in biological triplicates. *p < 0.05 in One-way ANOVA followed by Bonferroni’s test.

In order to gain more information and validate the predicted transcription factors and their involvement in the ROS-mediated inflammatory profiles in the context of Meth, as well as in Tat interactions, we characterized the transcription factor dynamics in macrophages treated as above. For that, we measured levels and translocation of the predicted transcription factors in Figure 7, in protein extracts and in cell cultures, by western blot and immunocytochemistry respectively (Figures 9, 10). More specifically, we focused on cJUN and cFOS, and NFkB pathway elements, and their key upstream signaling transactivation elements, the SRC kinase and the epidermal growth factor receptor EGFR (Fromm et al., 2008; Troib and Azab, 2015; Zhang et al., 2016).

In protein extracts, we found that Meth treatment caused an early enrichment, phosphorylation and translocation of cFOS and cJUN, as well as of NFkB (Figures 9, 10), detec**Table 1H** after stimulation. HIV Tat did not modify the levels or phosphorylation of cJUN and cFOS, as determined by western blot (Figure 9), but did facilitate their nuclear translocation (Figure 10). Regarding NFkB, Tat had a significant effect on its translocation, which did not occur when Meth was the stimulus (Figures 10E,F). The ROS scavenger NAC prevented the phosphorylation of cFOS and decreased NFkB levels. NAC also decreased Meth-induced translocation of cJUN (Figures 10A,B) and cFOS (Figures 10C,D), but enhanced the translocation of NFkB (Figures 9E,F).

The AP1 and NFkB upstream elements SRC kinase and EGFR were affected by Meth. EGFR levels and phosphorylation were increased by Meth, Tat and by NAC alone. However, these effects were not prevented when NAC was co-incubated with Meth. Total SRC was increased by Meth, Tat and NAC alone. NAC in the presence of Meth did not prevent the increase of total SRC, but completely blocked the SRC phosphorylation (Figures 9A,B).

**FIGURE 11:**
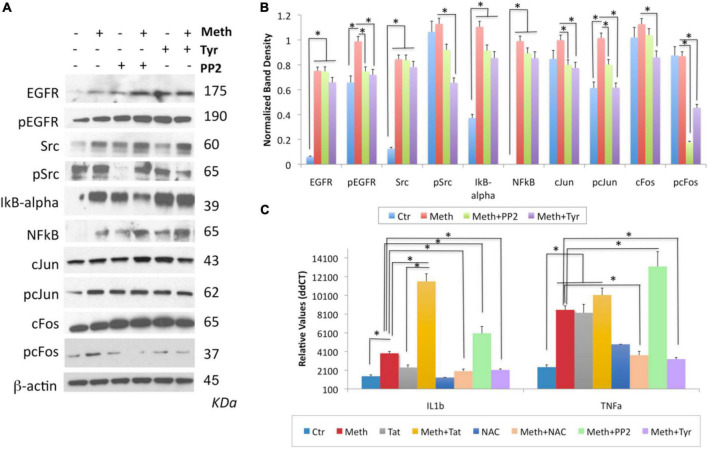
The contribution of the transactivation of EGFR and its downstream SRC kinase to the induction of IL1β and TNFα by Meth. The role of EGFR and Src was examined by incubation with pharmacological inhibitors, Tyrphostin (Tyr, 1 uM) and PP2 (1 uM), respectively, added 30 min after Meth (60 uM) was used as stimulus. Western blots were used to confirm the effect of these compounds on the levels of EGFR, pEGFR, SRC, pSRC, cJUN, pcJUN, cFOS and pcFOS, but also whether they have an impact on the IkB-alpha, NFkB pathway, using specific antibodies, all normalized internally with the detection of b-actin. **(A)** Representative western blots showing all the analyzed transcription factors and the examined conditions. Molecular weight of each band is shown on the right side of the panel. **(B)** Normalized band density was calculated in Image J, from 2-3 independent experiments. **(C)** Effect of EGFR and SRC inhibition, as well as of Tat and NAC, on the transcription of IL1β and TNFα. *p < 0.01 in indicated comparisons. Two-way ANOVA followed by Bonferroni’s test.

Following the characterization of ROS-regulated transcription factor dynamics, the transcription factors that were regulated by ROS in the context of Meth were pharmacologically blocked to test their impact in the transcription of two read-out genes selected based on RNAPol promoter binding and transcription upregulated in correlation with ROS. The genes were IL1β and TNFα, which have been extensively linked to inflammation and neurological outcomes in the context of substance use and HIV (Shapshak et al., 2011; Del Guerra et al., 2013; Coelho-Santos et al., 2015). We focused on AP1 (cJUN/cFOS) as transcription factors regulated by ROS in the context of Meth and their contribution to inflammatory gene transcription, via pharmacological blockage of cSRC phosphorylation by PP2, or blockage of EGFR phosphorylation by Tyrphostin (Tyr) (Figure 11). These blockers affect the downstream binding of AP-1 (cJUN/cFOS) to target gene promoters. Both PP2 and Tyr were added 30 min after Meth, to allow for ROS production, and then were maintained in the cultures for 2 h.

**FIGURE 12:**
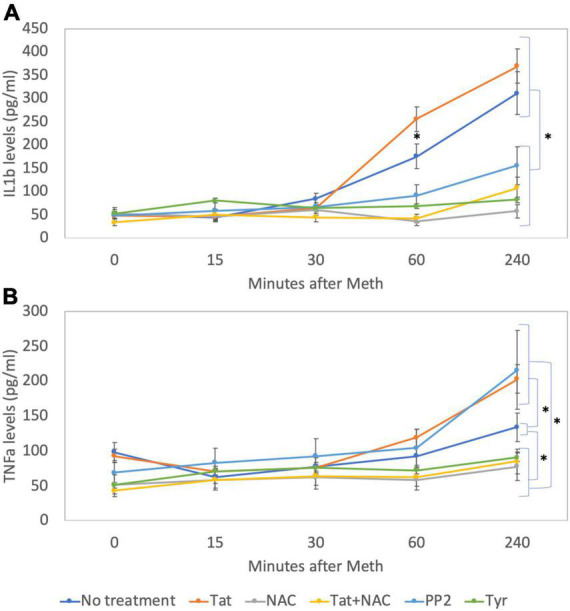
Levels of IL1β and TNFα on cell culture supernatants. By ELISA, levels of **(A)** IL1β and **(B)** TNFα were measured in cell culture supernatants at baseline and 15, 30, 60, and 240 min after addition of Meth (60 uM) to the cultures. The lines indicate cytokine levels upon the co-incubation with Tat (10 ng/ml), NAC (1 uM), Tyr (1 uM) and PP2 (1 uM). Results are the mean ± SD of triplicates, in 2 independent experiments. *p < 0.05 in repetitive measures ANOVA.

By western blot, we confirmed that Tyr decreased levels of pEGFR, pSRC, as well as pcFOS, and it blocked Meth-induced TNFα and IL1β transcription (Figures 11A-C), suggesting that the EGFR pathway is activated by Meth to induce these cytokines. On the other hand, PP2 decreased SRC phosphorylation in the absence of Meth (Figure 11A) but not in its presence (Figures 11A,B). Interestingly, PP2 increased Meth-induced IL1β and TNFα transcription (Figure 11C), suggesting that SRC phosphorylation may responds to EGFR but is not co-regulated by other Meth-induced ROS-dependent transcriptional factors, and is not directly involved in the effects of Meth. Importantly, the effects of PP2, as well as Tyr, on Meth-induced IL1β and TNFα protein secretion were detectable in cell culture supernatants 2 hours after stimulation (Figures 12A,B).

## Discussion

There is growing recognition of the effects of ROS on cell-signaling with functional consequences in homeostasis and pathology. Here we show that in the context of stimulant substances such as Meth, the increase in ROS production, particularly H_2_O_2_, is a significant evolutionary conserved component of promoter activity activation and transcriptional changes, which in innate immune cells, has implications in the inflammatory process (Hensley et al., 2000; Dickinson and Chang, 2011; Labunskyy and Gladyshev, 2013).

Given the overlap between Meth use and HIV infection, interactions at the molecular level must be considered in outcomes of neurocognitive aggravation. The HIV-1 Tat peptide is particularly relevant as a driver of interactions with Meth, due to its broad spectrum of actions in the cellular processes, but also for remaining detectable in the CSF and brain tissue of a significant fraction of virally suppressed subjects (Hudson et al., 2000; Henderson et al., 2019). HIV Tat also has the ability to directly induce ROS in macrophages, particularly H_2_O_2_, adding to the effect of Meth on ROS. Redundancy occurred in some genes induced by ROS, such as IL1β and TNFα. Yet, the majority of Tat-induced changes were non-redundant in comparison to Meth and were, for the most part, independent of ROS. Tat and ROS, independently led to early changes in RNAPol recruitment to promoters, indicating transcriptional activity. Several contrasting effects between Meth via ROS and Tat, including on NFkB translocation, confirm previous findings suggesting that Tat interferes with host transcriptional programs directly, counteracting some of the suppressive effects of Meth on gene transcription (Basova et al., 2020). We have previously shown that Tat can translocate to nucleus to regulate host cell gene transcription, particularly of inflammatory genes, and largely via Tata box-binding peptide (TBP), targeting early response gene promoters (Tjitro et al., 2018). The link between Tat and host chromatin, and the interactions of Tat with TBP, were further suggested in chromatin immunoprecipitation studies (Raha et al., 2005; Marban et al., 2011). Here, in a macrophage cell line system, we found genes with decreased promoter activity in the presence of Meth, in a ROS-dependent manner, but that were enhanced both by Tat alone or by treatment with Tat + Meth, in a ROS-independent manner. This interpretation comes from the fact that RNAPol and transcription were not modified by NAC in the context of Tat. Redundancy between Meth and Tat regarding effects of ROS may occur. However, the effects of Tat, and of Meth via ROS, indicate the activation of distinct paths, which may contribute to and enhance pathogenesis. In our system, we found that Tat can strongly downregulate the expression of anti-oxidant genes in innate immune cells, which are involved in detoxification and prevention of protein and DNA degradation involved in a number of conditions (Nathan and Cunningham-Bussel, 2013; He et al., 2017). In Meth exposure, oxidative stress plays a critical role in the induction of DNA damage and toxicity, which can be prevented by NAC (Zhang et al., 2012). In spite of the independent genomic effects between Meth and HIV-1 Tat, this viral peptide can exacerbate the damaging consequences of Meth-induced oxidative stress, while counteracting its effects on transcription.

We have focused on inflammatory genes upregulated by Meth. Yet, a number of genes were decreased by Meth in a ROS-dependent manner. The expression of STIM2 was recovered by NAC, but not by Tat. On the other hand, the decrease of TBP and CD81 were prevented by NAC as well as by Tat, in spite of the ability of Tat to increase ROS. This indicates that there is redundancy between Meth and Tat in effects but highlights the properties of Tat that bypass ROS regulation. The redundancy between the two stimuli does not mean that the regulatory triggers are the same. The use of NAC as a pharmacological tool was critical to make these distinctions.

IL1β and TNFα were among the genes that showed increased transcription triggered by Meth and prevented by NAC, indicating ROS-dependent Meth-induction, with a role in neuroinflammation. Tat also induced these genes, but this effect was not additive when in combination with Meth, but equally prevented by NAC (Supplementary Data).

Gene expression is regulated via transcription factors and regulatory mechanisms according to promoter characteristics, motif accessibility, transcriptional factor availability, phosphorylation and ability to translocate. We used computational predictive models to estimate regulators responsive to ROS in the context of Meth, using data on early RNAPol promoter activity. NFkB and AP-1 (cJUN/cFOS) were linked to the largest number of genes with significant changes, particularly among the ones with consequences to inflammation. NFkB is a transcription factor that is strongly associated with inflammation (Tak and Firestein, 2001). NFkB can be enhanced by IL1β and TNFα (Israël et al., 1989; Osborn et al., 1989), transcriptionally detectable in our system from 1-h post stimulation. However, RNAPol promoter activity was measured 15 minutes after stimulus to facilitate the identification of factors that result from ROS or direct stimulation, prior to the detection of cytokines. We discounted the contribution of NFkB in ROS-mediated gene upregulation, because Meth decreased its translocation. It is possible that NFkB is translocated at later time points, as a result of autocrine stimuli, and not directly attributed to ROS. These results suggest a mechanism that can explain the strong effect of Meth on transcriptional downregulation, which is counteracted by HIV-1 Tat (Basova et al., 2020). The in-depth examination of changes in the predicted factors indicated that cFOS is predominantly affected by ROS in the context of Meth. The contribution of this pathway to transcription was validated using the transcription of IL1β and TNFα.

ROS may signal transcription through the activation of Ca2 + -dependent cascades and activating early response transcription factors such as AP-1 (cJUN/cFOS) through SRC kinase, to affect a number of genes, including IL1β and TNFα (Ding et al., 1999, 2001; Guo and Wu, 2000; Roman et al., 2000; Chandrasekar et al., 2005; Hsieh et al., 2010; Lin et al., 2012; Yang et al., 2012; Samanta et al., 2014). The tyrosine kinases of epidermal growth factor receptor (EGFR) have also been described in association with the transduction cascade identified in our predictions, modulating cell proliferation, survival, adhesion, migration and differentiation (Malliri et al., 1998; Li et al., 2003). Regarding EGFR, its phosphorylation was increased by Meth. Yet, NAC in the context of Meth did not prevent EGFR increase in levels and phosphorylation. Tyrphostin, an EGFR inhibitor, decreased pSRC and pFOS, and decreased Meth-induced IL1β and TNFα. This suggests that although EGFR affects SRC and AP-1 downstream, with effects in the transcription of the inflammatory genes, the effects of ROS may bypass EGFR. SRC and AP-1 were involved in the transcription of IL1β and TNFα with contribution of ROS, since SRC phosphorylation was impaired by NAC. Moreover, the inhibition of SRC phosphorylation prevented cFOS phosphorylation. Intriguingly, the SRC inhibitor caused an increase in expression of IL1β and TNFα in the context of Meth. Thus, although ROS affected both cFOS and SRC phosphorylation directly, these factors cross-communicate to regulate the transcription of IL1β and TNFα in a ROS-dependent fashion, where SRC may serve to partially repress the effect of AP-1.

The single cell system we have used presents limitations imposed by immortalization and by the lack of additional cell types that are relevant to create the conditions triggered in the brain by Meth and HIV observed *in vivo*. For instance, a mouse model of cFOS deficiency has suggested that this transcription factor has a protective effect against Meth-induced neurotoxicity and dopaminergic dysfunction (Deng et al., 1999). On the other hand, cFOS can help identify molecular events happening in the innate immune population with specific relevance to the inflammation process. The relationship between ROS and cFOS has been previously described and linked to the contribution of astrocytes to tissue damage (Hsieh et al., 2010). In neurons, on the other hand, cFOS can be induced with the support of sympathetic input (Senba et al., 1993) and with implications to pain. Conversely, b-adrenergic antagonists that prevent anxiety and behavioral stress responses also ameliorate microglia activation while decreasing cFOS (Wohleb et al., 2011). cFOS is also involved in the potentiation of HIV-1 Tat-mediated activation of redox-sensitive brain regions in the context of Meth, with effects on TNFα and IL1β in striatum (Flora et al., 2003), suggesting implications to dopaminergic dysfunctions observed in HIV and Meth. Together, these studies point to the site and cell-specific role of cFOS and the AP-1 complex in regulating inflammation. Our study indicates that AP-1 in innate immune cells may be a key regulator of inflammatory factors aggravating neurological disorders in HIV and Meth users.

This is study is also limited to acute, short-term exposure to Meth and Tat simultaneously. The nature of Meth exposure in translational settings includes a number of tissue-specific complexities derived from multicellular effects, active infection, antiretrovirals and presence of other HIV proteins, in addition to regimen. Yet, this cell line *in vitro* system replicates effects we have previously identified in an *in vivo* model of neuroHIV (Tjitro et al., 2018; Basova et al., 2020), serving to isolate interactive effects in inflammatory cells.

**FIGURE 13:**
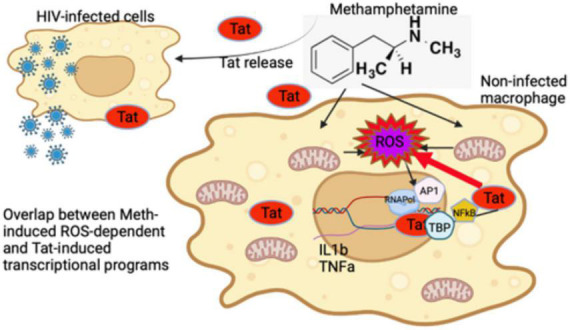
Early events in innate immune cells resulting from interactions between Methamphetamine and HIV Tat. Tat released from HIV infected cells can modify transcription of non-infected cells and enhance the effects of stimulant substances such as Meth. Meth affects gene transcription immediately largely *via* ROS. While Tat further increases ROS-production by suppressing antioxidant genes upregulated by Meth, it does not potentiate the effects of ROS, represented by induction of IL1β and TNFα genes *via* the activation of AP1 complex. The effects of Tat may bypass and add upon the effects of ROS induced by Meth, by activating other transcription factors, such as TBP and NFkB. As a result, an enhanced pro-inflammatory profile, resulting from a combination of ROS-dependent and -independent transcriptional factors, contribute to the pathogenesis of HIV in the context of Meth exposure.

The majority of the effects of Meth result in transcriptional suppression, yet sets of inflammatory genes appear to be specifically upregulated by Meth (Basova et al., 2020). Our innate immune cell system indicates that Meth acts selectively on early response innate immune genes by modulating RNAPol recruitment to promoters through both ROS-dependent and -independent mechanisms. ROS contributes to the upregulation of a significant fraction of inflammatory genes, including IL1β and TNFα, via phosphorylation and translocation of AP-1 components, bypassing EGRF. Although the activation of the SRC kinase is also responsive to ROS in the context of Meth, its effects are rather counteractive, partially repressing ROS effects on transcription of IL1β and TNFα. The HIV Tat peptide increases ROS, but it does not potentiate ROS signaling in inflammatory gene transcription. Instead, HIV Tat acts independently of ROS in large sets of host genes (Reeder et al., 2015; Tjitro et al., 2018), in many cases counteracting effects of Meth. The effects of Tat may occur by facilitating NFkB translocation and activating alternative transcription factors. We have previously shown that in THP1 cells, the effects of Tat on the activation of host gene promoters occurs directly and primarily through usage of TBP (Tjitro et al., 2018). The results here confirm TBP usage and suggest other alternative transcription factors that can bypass and enhance ROS signaling. Importantly, HIV-1 Tat may play a critical role enhancing oxidative damage in combination with Meth, by decreasing the expression of anti-oxidant genes and thereby suppressing the detoxifying machinery. Figure 13 shows a schematic illustration of these early events resulting from the interactions between Meth and HIV Tat in macrophages, resulting in inflammation.

## Data availability statement

The datasets presented in this study can be found in online repositories. The name of the repository and accession number(s) can be found below: NCBI Gene Expression Omnibus; accession number: GSE119015

## Author contributions

LB performed cell cultures, stimulations, western blots, computational analysis and PCRs. WV performed data analysis and curation. NB performed translocation studies. JN contributed to translocation and inhibitor studies. MM designed and overlooked the experiments, and obtained funding. All authors contributed to the article and approved the submitted version.
